# First-year nursing students’ initial contact with the clinical learning environment: impacts on their empathy levels and perceptions of professional identity

**DOI:** 10.1186/s12912-022-01016-8

**Published:** 2022-08-23

**Authors:** Qinghua Wang, Xiaohong Cao, Tianjiao Du

**Affiliations:** 1 Institute of Foreign Languages, China Medical University, No. 77 Puhe Road, Shenyang North New Area, Shenyang, Liaoning Province People’s Republic of China; 2 School of Marxism, China Medical University, No. 77 Puhe Road, Shenyang North New Area, Shenyang, Liaoning Province People’s Republic of China; 3 School of Medical Humanities, China Medical University, No. 77 Puhe Road, Shenyang North New Area, Shenyang, Liaoning Province People’s Republic of China

**Keywords:** First-year nursing students, Clinical learning environment, Empathy levels, Professional identity perceptions, Initial contact, Mixed-methods study

## Abstract

**Background:**

Research shows that the clinical learning environment can affect medical learners’ levels of empathy and formation of professional identity. However, few studies examined the impacts of early exposure to the clinical learning environment on first-year nursing students’ empathy levels and professional identity perceptions.

**Aim:**

This study aimed to explore effects of initial contact with the clinical learning environment on first-year nursing students’ empathy levels and perceptions of professional identity.

**Methods:**

This is a mixed-methods study conducted in a medical university and its affiliated hospital in Northeast China. For quantitative analysis, 220 first-year nursing students finished Interpersonal Reactivity Index (IRI) twice before and after their five-day clinical placement in the hospital in June, 2021. Paired samples t tests were used to explore the changes in first-year nursing students’ cognitive empathy, affective empathy and total empathy levels as measured by IRI before and after the clinical placement. For qualitative analysis, 15 first-year nursing students’ diary recording their clinical learning experiences were analyzed. An inductive thematic analysis approach was adopted to extract themes from the content on professional identity in nursing students’ diary.

**Results:**

After the five-day clinical placement, first-year nursing students’ cognitive empathy, affective empathy and total empathy levels all increased. Five themes emerged regarding nursing students’ perceptions of professional identity: (1) *Love for the nursing profession;* (2) *Multiple roles nurses play;* (3) *Personal characteristics a good nurse needs to have;* (4) *Deeper understanding of the nursing profession;* (5) *New understanding of the relationships between patients and nurses, between patients and doctors, and between doctors and nurses.*

**Conclusions:**

First-year nursing students’ initial contact with the clinical learning environment helped them enhance empathy levels and shape professional identity. Nursing educators may consider providing nursing students with opportunities of early exposure to the clinical learning setting to cultivate their empathy and develop their professional identity.

**Supplementary Information:**

The online version contains supplementary material available at 10.1186/s12912-022-01016-8.

## Background

A harmonious nurse-patient relationship, which should be established based on mutual understanding as well as mutual trust, is essential for providing high-quality nursing services [1]. Good communication, in both verbal and non-verbal forms, is the basis of developing and maintaining a harmonious nurse-patient relationship [2].

Research shows that empathy levels of nurses exerted a significant impact on nurse-patient communication and nursing care quality [3, 4]. Empathy, as a multidimensional construct, comprises both cognitive and affective aspects. Cognitive empathy refers to an individual’s ability to understand another person’s experiences and the ability to communicate this understanding, while affective empathy entails an individual’s concern for another person’s feelings [5]. In the nurse-patient relationship, compared with affective empathy, cognitive empathy has been demonstrated to play a relatively more important role [6]. Research shows that empathy is an attribute that develops over the course of one’s life and is also a malleable construct that can be nurtured [6, 7].

In nursing education, different approaches have been developed to cultivate nursing students’ empathy, such as communication skills training [8], empathy role-playing program [9], Virtual Dementia Tour [10], immersive digital story intervention [11] and expert-patient teaching [12]. In addition to these interventions, studies show that the clinical learning environment can also affect medical learners’ levels of empathy [13]. However, we found few studies examining the impacts of early exposure to the clinical learning environment on first-year nursing students’ empathy levels. Therefore, the first aim of the present study was to explore this issue.

Besides empathy levels, the professional climate of the clinical learning environment may also affect nursing students’ formation of professional identity. Students will view nurses and doctors in the clinical environment as role models. Literature shows that attitudes and behaviors demonstrated by role models play an essential role in shaping medical learners’ professional values and professional identity [14]. In the clinical learning environment, not only can nursing students witness how nurses and doctors communicate with patients, but they can also have a direct contact with patients. Such communication between healthcare providers and patients, also known as health communication (HC), is a kind of professional communication which is found to be critical for patient compliance and patient safety as well as professional identity [15–17]. In the clinical learning environment, besides communicating and learning with their clinical nursing teachers, nursing students also have the opportunity to communicate with patients, both verbally and non-verbally. Although some previous studies have already explored different aspects of nursing students’ experiences in the clinical setting [18–20], how early exposure to the clinical environment helps shape nursing students’ professional identity was rarely explored. Therefore, the second aim of the present study was to examine the impacts of initial contact with the clinical learning environment on first-year nursing students’ perceptions of professional identity.

## Methods

### Study design

This study adopted a mixed-methods design including both quantitative and qualitative analyses. For quantitative data, a pretest–posttest quasi-experimental design was used to explore the change in first-year nursing students’ empathy levels after their five-day clinical placement in the affiliated hospital of the authors’ medical university. For qualitative data, thematic analysis approach was employed to extract main themes regarding first-year nursing students’ development of professional identity by referring to their diaries.

### Setting

This study was conducted in a key medical university and its affiliated hospital in Northeast China. In the second semester of the first academic year, nursing students took a course called *Introduction to Nursing* (For course details, please refer to the additional file 1)*.* This introductory course for first-year nursing students aims to familiarize students with the basic theories in nursing and help students grasp the general framework of the nursing discipline. In order to strengthen the connection between theory and practice, and reduce the drawbacks of the traditional curriculum brought by “the separation between basic theory acquisition through didactic lectures and hands-on experiences through clinical practice”, the authors’ medical university arranged a five-day clinical placement in its affiliated hospital for first-year nursing students at the end of *Introduction to Nursing* course. All first-year nursing students (9 classes, 270 students) in the medical university took the *Introduction to Nursing* course and participated in the following five-day clinical placement in the hospital.

The clinical placement started at 8:30 and finished at 16:00 each day for five consecutive days (21^st^ June 2021 – 25^th^ June 2021). First-year nursing students were allocated into 26 divisions, i.e. Respiratory Department, Cardiovascular Department, Cardiovascular ward, Digestive Department, Hematology Department, Neurology Department, Nephrology Department, Endocrinology Department, Rheumatology and Immunology Department, Infectious Disease Department, Hepatological Surgery Department, Pancreatic Surgery Department, Vascular Thyroid Surgery Department, Gastrointestinal Surgery Department, Orthopedics Department, Neurosurgery Department, Thyroid Surgery Department, Urinary Surgery Department, Cardiac Surgery Department, Oncological Surgery Department, Motor Joint Ward, Thoracic Surgery Department, Interventional Ward, Gynecology Ward, Otolaryngology Department and Dermatology Department. For each division there was a head nurse, and each clinical nursing teacher (a clinical nurse working in the affiliated hospital of the authors’ medical university) supervised 1–2 nursing students.

### Aims and contents of the clinical placement

The aims of the clinical placement attached to the course *Introduction to Nursing* were: (1) to enhance nursing students’ perceptual understanding of the role and functions of nursing work; (2) to enable nursing students to apply the acquired basic theoretical knowledge to real clinical practice; (3) to help nursing students shape the ideal and values of nursing profession; (4) to instill the concept of patient-centered services and cultivate nursing students’ professional identity; (5) to help nursing students gain a deeper understanding of the relationships between doctors and nurses, between doctors and patients, and between nurses and patients in the real clinical environment; (6) to let nursing students observe and evaluate nursing professional behaviors based on the standards/code for a qualified nurse; (7) to let nursing students experience the medical work environment; (8) to let nursing students do some work for patients under the guidance of clinical nursing teachers. The clinical placement includes 11 sessions: the nursing profession and a nurse’s role, health and disease, needs and culture, growth and development, stress and adaptation, scientific thinking and clinical decision making, nursing procedures, nursing theories, health education, legal issues in nursing and routine clinical nursing work (For details, please refer to the Additional file 2).

### Participants and data collection

To examine the change in first-year nursing students’ empathy levels before and after their five-day period of clinical placement in the hospital during which they had an initial contact with the clinical learning environment, we invited all first-year nursing students in our university to participate. Among the 270 nursing students invited, 220 students (180 females, 40 males, Mean Age: 18.66 years, Standard Deviation: 0.77) agreed to participate (response rate: 81.5%). We used an electronic questionnaire platform called Juanxing Wen to collect the quantitative data and every nursing student who agreed to participate filled out questionnaires online twice just before and immediately after the five-day clinical placement in June, 2021.

To examine impacts of the initial contact with the clinical learning environment on first-year nursing students’ professional identity perceptions, before the clinical placement in early June, 2021, the researcher (the first author WQ) recruited nursing students who would like to keep a diary about their experiences and feelings during the clinical placement. Because this was not a required task in the curriculum and the extra work of keeping a diary during the busy period of clinical placement would certainly add to students’ workload, only 15 first-year nursing students (all females) volunteered to participate in the diary study. After the clinical placement, the researcher (the first author WQ) asked these 15 first-year nursing students to hand in an electronic version (i.e. in Word format) of the diary in July, 2021.

### Measures

#### Empathy

Nursing students’ empathy levels before and after the clinical placement were measured with Interpersonal Reactivity Index (IRI) developed by Davis [21]. IRI included 28 items falling into four subscales: Fantasy Scale (FS: 7 items), Perspective Taking (PT: 7 items), Empathic Concern (EC: 7 items) and Personal Distress (PD: 7 items). The two subscales of Fantasy Scale and Perspective Taking constituted cognitive empathy (FS + PT: 14 items) and the two subscales of Empathic Concern and Personal Distress constituted affective empathy (EC + PD: 14 items) [21]. Each item was scored on a five-point Likert scale from 0 (does not describe me well) to 4 (describes me very well). After the negatively worded questions were reverse scored, the total score of the four subscales was calculated to indicate nursing students’ overall levels of empathy. The sum score of the two subscales of FS and PT was calculated to indicate nursing students’ levels of cognitive empathy, and the sum score of the two subscales of EC and PD was calculated to indicate nursing students’ levels of affective empathy. IRI Chinese version was used in health profession students and demonstrated satisfactory reliability in previous research [22]. In the current study, Cronbach’s alpha coefficients for total empathy, cognitive empathy and affective empathy were 0.731, 0.635 and 0.642, respectively in the pre-test (before the clinical placement) and were 0.760, 0.689 and 0.704, respectively in the post-test (after the clinical placement).

#### Professional identity

Impacts of the initial contact with the clinical learning environment on first-year nursing students’ perceptions of professional identity were analyzed by referring to the content in nursing students’ diary which recorded their five-day clinical learning experiences in the hospital (what they did, what they saw, what they heard and what they learnt) and their feelings (how they felt, what they thought and their reflections) during the five-day clinical placement. Nursing students kept a written diary in Chinese during the five-day clinical placement since most students do not speak English or write in English in daily life. After the clinical placement, nursing students handed in an electronic version of the diary as requested by researchers for convenience of data analysis.

### Data analyses

#### Quantitative analysis

For quantitative data, IBM SPSS statistics (IBM Corp., Armonk, NY, USA) version 22.0 was used to conduct the descriptive statistics analyses of the participants’ socio-demographic characteristics, means and standard deviation of the cognitive empathy subscale, the affective empathy subscale as well as the total empathy scale, and the reliability analysis of the scale and subscales in the pre-test and in the post-test. Paired-samples t test was adopted to compare scores of the cognitive empathy subscale, scores of the affective empathy subscale and scores of the total empathy scale between the pre-test and the post-test.

#### Qualitative analysis

For qualitative data, a thematic analysis was conducted on the content regarding professional identity in nursing students’ diary. Based on the framework of grounded theory [23], the present study adopted an inductive thematic analysis approach developed by Braun and Clarke [24]. First, researchers identified keywords and phrases relating to the concept of professional identity independently. These keywords and phrases form the basis for the coding scheme. Second, independent researchers met, pooled the keywords and phrases each collected, looked for patterns and recurring information, and developed a “thematic map” together. Then emergent themes in the initially generated “thematic map” were compared and further refined, and the finalized overarching themes were agreed upon by all researchers after several rounds of discussion until a consensus was reached.

### Rigor

For quantitative analysis, we used Interpersonal Reactivity Index (IRI) to measure nursing students’ levels of cognitive empathy, affective empathy and total empathy before and after the clinical placement. IRI is one of the most world-widely used scales to measure the construct of empathy and its facets, and IRI Chinese version has demonstrated sound psychometric properties among medical learners in previous studies [22, 25, 26]. In the current research, the reliability of IRI Chinese version was acceptable among nursing students as indicated by Cronbach’s internal consistency coefficients. For qualitative analysis, nursing students’ diary about their clinical learning experiences was written in Chinese since it is convenient and time-saving to keep a diary in one’s mother tongue. The first author of this manuscript WQ, who got the Master’s Degree in English and the Doctor’s Degree in Medicine, and proficient in both Chinese and English, translated the diary content from Chinese into English. In order to reduce bias, WQ then asked another PhD scholar (not an author of this manuscript), who has overseas study experiences and also proficient in both Chinese and English, to translate the English version of diary content backwards into Chinese. After that, the two Chinese versions of diary content (i.e. the original version written by nursing students and the translated Chinese version by the PhD scholar with overseas study experiences) were compared. Once there was discrepancy between the two versions, discussions were held between the two translators and the research team members of this study, and if needed, the opinions of the nursing student who wrote the diary were also sought for clarity. The final English version of nursing students’ diary was approved by all parties involved including research team members, translators and nursing students after rounds of discussions. All 15 first-year nursing students’ diary content about professional identity was analyzed, data saturation was achieved after we analyzed 13 first-year nursing students’ diary content as no more new themes regarding professional identity emerged.

### Ethical approval

This study was approved by the authors’ affiliated medical university and complied with the code of the Declaration of Helsinki. The purpose of the study (i.e. for research only) was explained beforehand and nursing students were assured that participation was voluntary. Every nursing student who agreed to participate read the details in an electronic version of the informed consent form and signed it online.

## Results

### Changes in nursing students’ empathy levels before and after the clinical placement

Table 1 shows the changes in first-year nursing students’ levels of cognitive empathy, affective empathy and total empathy before and after their five-day clinical placement in the hospital. Results of paired samples t tests reveal that after the clinical placement, nursing students’ cognitive empathy level was significantly higher (34.20 ± 5.55 vs 36.97 ± 5.09, t = -7.84, *p* < 0.001), and nursing students’ affective empathy level was also significantly higher (32.20 ± 5.40 vs 33.35 ± 5.84, t = -3.29, *p* < 0.01). There was a significant increase in first-year nursing students’ total empathy level after they took the five-day clinical placement (66.39 ± 9.09 vs 70.32 ± 8.93, t = -9.35, *p* < 0.001).

**Table 1:** Comparisons of first-year nursing students’ levels of cognitive empathy, affective empathy and total empathy before and after the clinical placement

Study variables	Before the clinical placement (M ± SD)	After the clinical placement (M ± SD)	t	*P*
Cognitive empathy	34.20 ± 5.55	36.97 ± 5.09	-7.84	< 0.001
Affective empathy	32.20 ± 5.40	33.35 ± 5.84	-3.29	< 0.01
Total empathy	66.39 ± 9.09	70.32 ± 8.93	-9.35	< 0.001

*N* = 220; *M* ± *SD* mean ± standard deviation

### Thematic analyses of nursing students’ perceptions of professional identity after their initial contact with the clinical environment

#### Love for the nursing profession

Nursing students expressed their deeper feelings and love for the nursing profession after their first contact with the clinical learning environment. They also presented the idea that they had a sense of mission and felt more enthusiastic towards the nursing profession upon seeing in person what nurses did for patients in hospital.

“…seeing my teacher nurse change trocar skillfully while answering patients’ questions kindly, I seem to love my major more than when I applied for it after the college entrance examination….helping others makes one’s own life meaningful and I’ve got a sense of mission towards this profession…”–-N1.

“…I was assigned to the vascular and thyroid department and this is the first time I am learning in a clinical environment after entering the university. I have a lot of experiences that I cannot get through learning from books in the classroom…What I see, what I hear and what I do in the hospital gave me a better understanding of the nursing profession and made me feel more passionate and enthusiastic towards the profession.”–-N7.

“Saving one’s life, alleviating one’s pain, seeing one coming in with a sad face and leaving with a smiling face, there is no way to describe my feelings. Employees in most of other work fields are happy to see more customers coming in, but we really hope that patients recover soon and say goodbye to us…”–-N3.

#### Multiple roles nurses play

Nursing students expressed the idea that before the clinical placement, they thought that nurses had only one role, i.e. to look after patients, but after their initial contact with the clinical learning environment, students said that they had a deeper understanding of multiple roles nurses played in real clinical environment. Besides caregivers, nurses are also decision-makers, planners, communicators, coordinators, consultants, educators, health facilitators and researchers.

“…My clinical nursing teacher had planned to start with an acid suppressant and a liver-protecting drug, then an anti-inflammatory drug and finally a painkiller. However, the patient complained of unbearable pain and asked for painkillers first. My nurse teacher agreed to the patient’s request and gave him the painkiller… I think nurses are decision-makers as well as planners, and they can adjust their nursing plan based on their professional knowledge and their own judgment of the situation…”–-N9.

“For patients and their families, nurses will provide information about dos and don’ts to ensure patients’ safety and recovery. In addition to giving advice, nurses also ask for patients’ and their families’ opinions on healthcare issues… I think besides caregivers, nurses also play roles of communicators, coordinators, consultants, educators,and health facilitators.–-N2.

“During the rest time at noon, nurses will read some books and watch videos about nursing work to increase their professional knowledge and improve their ability. Doing excellent nursing work requires life-long learning, and nurses also need to do research…”–-N5.

#### Personal characteristics a good nurse needs to have

In nursing students’ diaries, personal characteristics such as empathy, sense of responsibility, altruism, compassion, patience, respectfulness, accountability, collaboration and pursuit of excellence are frequently mentioned. First-year nursing students stated that besides professional knowledge and skills, a good nurse is also supposed to have these personal characteristics.

“…a good nurse should be empathic and have a strong sense of responsibility, so that she can stand in patients’ shoes and understand what they really need…”–-N7

“Besides the collaboration between doctors and nurses, I think the relationship between nurses and patients as well as patients’ families are also cooperative, the respect between them are mutual, and the goals of them are the same: to get the best medical service for the patients…”–-N11.

“…patients suffer from disease, so they and their families are under great stress…In addition to physical pain, patients may also be afflicted by negative emotions such as anxiety and depression. I think good nurses are also good and patient listeners. They listen to their patients with compassion and provide emotional support…”–-N6.

“Nurses may encounter different new problems in daily work and they need to constantly learn to solve these problems. During break time, I often see my clinical nursing teacher flicking over books and this really gives me encouragement. I think, on the way to success and excellence, there is no shortcut…”–-N14.

#### Deeper understanding of the nursing profession

First-year nursing students expressed the idea that nursing work is much more complicated than they had expected. Students said that during their clinical placement, they had a deeper understanding of the nursing profession, knew what nurses actually do in real clinical environment and the critical role of the nursing station in the hospital.

“It was not until this clinical placement that I realized nursing work was indeed different from what I had imagined. Before the clinical placement, I always thought that the work of nurses was only simple operations, such as giving injections, changing dressings and drawing blood. However, I saw that the work in the inpatient department also include admission and discharge guidance, explaining patients’ conditions to their families, stabilizing patients’ emotions…”–-N10.

“If we compare nursing work to a house, the excellent theoretical knowledge and practical skills are like reinforced concrete, and empathy, kindness and warmth are like furniture, both are essential for a comfortable house. To provide good medical service to patients, nurses need to have both outstanding professional skills and empathic characters…”–-N4.

“…it is said that the work of a nurse is very heavy, some even say it is humble. After several days of observation, I felt that a small nursing station seemed to be a transportation hub. Newly admitted patients go to the nursing station for guidance, inpatients go to the nursing station for help, patients’ families go to the nursing station for information, doctors go to the nursing station for confirmation…The small nursing station acted as a contact hub in the hospital, which is indispensable…”–-N8.

#### New understanding of the relationships between patients and nurses, between patients and doctors, and between doctors and nurses

Some nursing students mentioned in their diaries that before the clinical placement, they had worried about the relationship between nurses and patients since the intense relationship between healthcare professionals and patients is frequently depicted on the media. After contacting with the real clinical learning environment, first-year nursing students expressed their new thoughts on the relationships between patients and nurses, between patients and doctors, and between doctors and nurses.

“…before the clinical placement, I was anxious about the nurse-patient relationship, and it seems to me that daily nurse-patient conflict is inevitable. However, during my five-day placement, I did not encounter any nurse-patient conflict and I think that this is not due to good luck, nor the patients and their families are tolerant. When I see my clinical nursing teacher’s attitude when communicating with patients and their families, I understand that a good nurse-patient relationship requires joint efforts of both sides, patients and their families’ understandings as well as nurses’ patience…”–-N8.

“…prior to this clinical learning experience, I always thought that the relationship between a doctor and a nurse is kind of between a superior and a subordinate, but during the clinical placement, I knew this is not the case. Doctors and nurses need to communicate, to collaborate, to cooperate for the same goal, i.e. to provide good medical care services for the patients. For example, a doctor may give a wrong medical order, and before the execution of the order, a nurse will check the doctor’s advice based on her own professional knowledge and if she has any doubts about the order, she would communicate with the doctor first…”–-N4.

“…I think the relationships between doctors and patients, between nurses and patients, and between doctors and nurses are the same in essence. They need to communicate with each other, to cooperate with each other, and to understand each other. Giving excellent medical services and receiving excellent medical services, this is in itself a mutual trade, so doctors, nurses and patients are like a community of shared interests. My clinical learning experience makes me believe that a harmonious relationship between patients and healthcare professionals can be built, conflicts and misunderstandings can be avoided…”–-N12.

## Discussion

The present study used both quantitative analysis and qualitative analysis to explore the impacts of first-year nursing students’ initial contact with the clinical learning environment on their levels of empathy and perceptions of professional identity. The results show that after the five-day clinical placement, nursing students’ levels of cognitive empathy, affective empathy and total empathy all increased, and themes extracted from qualitative analysis of the content in nursing students’ diary shed light on understanding the impacts of students’ first contact with the clinical learning environment on their perceptions of professional identity. These findings have important implications for nursing education.

In the past, according to the traditional nursing curriculum of our institution, nursing students will not have the opportunity to study in a clinical learning environment until their third academic year. In the first two academic years, nursing students acquire theoretical knowledge through didactic lectures and get hands-on experiences in laboratory courses. This late exposure to the clinical learning environment may lead to some problems. First, although nursing students can get a general idea about nursing work and nursing profession through classroom learning, their understandings are still superficial, especially when the subtle relationships among patients, doctors and nurses are concerned [27]. Second, whereas nursing students can obtain hands-on experiences in laboratory courses, the feelings they get when doing operations on models of human body or on simulated patients are quite different from what they feel when facing real patients in the clinical environment [28]. Third, only through classroom learning of professional knowledge and skills, nursing students’ empathy may not be well nurtured since they cannot get in touch with real communication between healthcare providers and patients [29]. In view of these drawbacks, our institution implemented the curriculum reform and arranged the five-day clinical placement following the course *Introduction to Nursing* in the affiliated hospital for nursing students in their second semester of the first academic year. The findings of our study indicate that early exposure to the clinical environment is helpful in fostering first-year nursing students’ empathy and in shaping their professional identity.

Empathy refers to the ability to stand in others’ shoes, to understand how they feel, to share their emotions and to communicate the shared feelings [6]. In the nursing field, empathy is found to be an important element in building a harmonious relationship between patients and nursing staff, which is essential for patient-centered care [28–30]. The English word “empathy” was first used in the 1920s by an American psychologist E.B.Titchener. According to Titchener’s theory, empathy stemmed from a sort of physical imitation (motor mimicry) of the distress of another, which then evokes the same feelings in oneself. This kind of motor mimicry is an instinct which can be observed in one-year-old toddlers and although it fades from toddlers’ repertoire at about two and a half years, the stimuli in the environment still play a critical role in developing one’s empathy [31]. In the clinical learning environment, the stimuli such as seeing patients suffering from illnesses and pain, observing how doctors and nurses treat patients and their families (i.e. by seeing how others react when someone else is distressed), and by communicating with patients themselves, first-year nursing students developed a repertoire of empathic response which helped enhance their empathy levels. This may explain the reason why our study found that after the clinical placement, nursing students’ cognitive empathy, affective empathy and total empathy all improved. An interesting finding of our study is that early exposure to the clinical learning environment exerted a larger impact on first-year nursing students’ cognitive empathy (34.20 ± 5.55 vs 36.97 ± 5.09, t = -7.84, *p* < 0.001) than on their affective empathy (32.20 ± 5.40 vs 33.35 ± 5.84, t = -3.29, *p* < 0.01) as demonstrated in Table 1. Although some previous research found that in the nurse-patient relationship, cognitive empathy played a relatively more important role than affective empathy [6], few studies have yet examined how immersing in the clinical learning environment where nursing students can get in contact with real communications between nurses and patients impacted nursing students’ cognitive empathy and affective empathy differently. The finding of the present study on this issue needs to be further explored and verified in future studies.

Thematic analyses reveal that learning in a clinical environment and having a direct contact with patients and healthcare professionals exerted a significant impact on first-year nursing students’ perceptions of professional identity. Through reflecting on what they saw and what they did during the clinical placement, students got a deeper understanding of the nursing profession and multiple roles nurses play, knew what personal characteristics a good nurse needs to have, and had a new understanding of the relationships between patients and healthcare professionals. Nursing students expressed the idea that their clinical learning experiences deepened their love for the nursing profession and strengthened their determination to learn nursing. Professional identity entails an individual’s conception of what it means to be and act as a professional [32]. Nursing professional identity involves the internalization of core values and perspectives which are recognized as integral to the art and science of nursing [33]. The formation of professional identity is a social process that develops in interactional relationships and in professional contexts [34]. In the clinical learning environment during the placement, first-year nursing students developed professional self-concept of attributes, beliefs, values, attitudes, motivations through observing how nurses communicated with patients and their families, how nurses communicated with doctors, and also through interacting with patients and nurses themselves. By reflecting upon and interpreting the experiences gained in the professional setting, nursing students gradually internalized the core values, characteristics, code of ethics, moral principles and norms of the nursing profession, which helped shape their professional identity. Literature shows that nursing students’ perceived professional identity has a direct relationship with student retention in the nursing program and is an important factor that affects whether they would choose nursing as their future career after graduation [35, 36]. Research indicates that first year of the nursing program is a critical period during which some students may decide to leave the nursing course [37]. The finding of our study that a short period of five-day clinical placement is helpful in shaping first-year nursing students’ professional identity is encouraging and can provide insights for nursing educators on identifying effective ways to retain students in the nursing program and reduce the attrition rate.

### Limitations and future directions

This mixed-methods study has several limitations. First, this study was conducted in only one medical university and its affiliated hospital in China, so generalizations of the conclusions should be made with caution and future multi-institutional research in different cultures is recommended. Second, for quantitative analysis, self-administered questionnaires were used to collect data, so there may be response bias and social desirability bias. Third, for qualitative analysis, only 15 female first-year nursing students’ diaries were available, but data saturation was achieved after we analyzed 13 nursing students’ diary content with the inductive thematic analysis approach. Fourth, the period of clinical placement was relatively short, covering only five consecutive days. It is recommended that future studies use a larger sample including both female and male nursing students to examine the effects of a longer period of early exposure to clinical practice on nursing students’ development of empathy levels and formation of professional identity.

## Conclusions

The present study explored impacts of first-year nursing students’ initial contact with the clinical learning environment on their empathy levels and professional identity perceptions. Results show that early exposure to the clinical setting in the first academic year, even for a short period of time, can help cultivate nursing students’ empathy and shape their professional identity. Nursing educators may consider providing nursing students with opportunities of immersing in the clinical learning environment early in their first academic year to help them enhance empathy levels and develop professional identity.

## Supplementary Information

**Additional file 1.** Introduction to Nursing.

**Additional file 2. **Contents of the clinical placement.

## Data Availability

The datasets used and/or analyzed in the present study are available from the corresponding author on reasonable request.

## Abbreviations

HC: Health communication
IRI: Interpersonal Reactivity Index
FS: Fantasy Scale
PT: Perspective Taking
EC: Empathic Concern
PD: Personal Distress
