# Supplementary material for: First-year nursing students’ initial contact with the clinical learning environment: impacts on their empathy levels and perceptions of professional identity
Source: BMC Nurs. 2022 Aug 23;21:234. doi: 10.1186/s12912-022-01016-8 (PMC9400203; doi:10.1186/s12912-022-01016-8)
Supplement: Supplementary file 1 — Additional file 1. Introduction to Nursing. [file 12912_2022_1016_MOESM1_ESM.docx]

*Introduction to Nursing*

(Theoretical lectures: 27 hours in total)

Chapter 1: Development and basic concepts of nursing

Chapter 2: Health and disease

Chapter 3: Need and care

Chapter 4: Culture and nursing

Chapter 5: Physical and mental development in the course of life

Chapter 6: Stress theory and its application in nursing

Chapter 7: Nursing process

Chapter 8: Nursing theory and model

Chapter 9: Scientific thinking method in nursing and decision-making

Chapter 10: Legal issues in the nursing profession

Chapter 11: Career planning in the nursing profession
