# Supplementary material for: First-year nursing students’ initial contact with the clinical learning environment: impacts on their empathy levels and perceptions of professional identity
Source: BMC Nurs. 2022 Aug 23;21:234. doi: 10.1186/s12912-022-01016-8 (PMC9400203; doi:10.1186/s12912-022-01016-8)
Supplement: Supplementary file 2 — Additional file 2. Contents of the clinical placement. [file 12912_2022_1016_MOESM2_ESM.docx]

**Additional Table 1** Contents of the clinical placement

| **Session** | **Topics** | **Main contents** |
| --- | --- | --- |
| 1. | The nursing profession and a nurse’s role | -Understanding the role of a nurse  -Knowing requirements of a qualified nurse  -Knowing the scope of nursing work |
| 2. | Health and disease | -Understanding a patient’s role  -Understanding a nurse’s role in health promotion |
| 3. | Needs and culture | -Using the theory of basic human needs, taking clinical patients as an example, analyzing the basic human needs and observing how nurses satisfy the needs of patients  -Using the cultural nursing theory and observing how nurses help patients adapt to the hospital cultural environment |
| 4. | Growth and development | -Using the psychological and social development theory, analyzing stages of patients’ growth and observing how nurses provide personalized care |
| 5. | Stress and adaptation | -Using the stress theory, observing and analyzing patients’ response, adaptation and coping to stress  -Observing how nurses help patients cope with stress  -Using the acquired knowledge to try to help patients cope with stress  -Observing and analyzing nurses’ work pressure and their coping strategies |
| 6. | Scientific thinking and clinical decision making | -Using the knowledge and skills acquired to reflect on and summarize the observed clinical problems  -Identifying types and patterns of clinical nursing decisions  -Observing how clinical nursing teachers use critical thinking to make clinical nursing decisions, solve complex problems in nursing practice and provide high-quality nursing services for patients |
| 7. | Nursing procedures | -Observing how clinical nursing teachers apply nursing procedures to provide nursing services for patients |
| 8. | Nursing theories | -Using Orem self-care theory, taking clinical patients as examples, and conducting case analysis of patients’ self-care ability, therapeutic self-care needs and self-care defects |
| 9. | Health education | -Getting familiar with the content, method and procedure of health education  -Observing the health education activities of clinical nursing teachers |
| 10. | Legal issues in nursing | -Using the acquired knowledge to observe and analyze the existing or potential legal issues in clinical nursing work  -Observing how clinical nursing teachers prevent legal risks |
| 11. | Routine clinical nursing work | -Learning basic nursing and specialized nursing techniques  -Observing and learning to use some common clinical equipment  -Getting familiar with the procedure of admission and discharge of patients, the writing of nursing documentation, the form and content of nursing shift and nursing rounds, and observing how nurses carry out doctors’ medical orders |
